# Guest editorial for the special issue: ECPA 2021

**DOI:** 10.1007/s11119-022-09966-4

**Published:** 2022-10-31

**Authors:** J. Bendig, Y. Li

**Affiliations:** 1 Insitute for Bio- and Geosciences, Research Centre Juelich, Juelich, Germany; 2 College of Mechanical and Electrical Engineering, Shihezi University, Shihezi, China

The 13th European Conference on Precision Agriculture, ECPA 2021, held in Budapest, Hungary in July 2021, was held in hybrid-format due to the then still very significant COVID-19 pandemic. Over 105 stimulating papers were presented, as well as many posters. The presentations covered a breadth of topics centred around the general theme of precision agriculture such as precision crop protection, monitoring of crop biochemical and biophysical variables as well as biotic and abiotic stress, progress in variable rate application and irrigation techniques, an increased use of machine learning techniques and other topics. The Precision Agriculture journal was pleased to invite extended manuscripts from ECPA 2021 for a special issue that represent a cross section of the research presented at the conference. Ten contributions appear in this special issue submitted from authors coming from seven different countries and covering several crops such as wheat, maize, cotton, tomato, wine and orchard trees. Many contributions included proximal and/or remote sensing techniques from ground- (Gee et al., Castro et al., Gobbo et al., Marani et al., Straub et al.), drone- (Lacerda et al.) or satellite-based sensors (Lacerda et al., Sandonis-Pozo et al., Pelta et al.), mainly focussing on exploiting images, spectral information or combining structural data derived from 3D point clouds. Lacerda et al., Karpinski et al. and Gobbo et al. examined variable rate application of irrigation, pesticide and fertilizer. Castro et al. looked into modelling leaf area index with deep learning. Gee et al. examined crop stress introduced through weed pressure. Vona et al. examined soil types within Hungary. Pelta et al. focused on the irrigation regime at the plot level through remote sensing and artificial intelligence. Marani et al. conducted semantic segmentation of natural images in viticulture based on semi-supervised learning methods. Straub et al. calculated tree models with 3D point clouds for estimating pruning points for meadow orchard trees.

This special issue was made possible through a collaborative effort of the Editors in Chief, Dr John Stafford, Dr Yang Li, and Dr. Juliane Bendig. We thank the committee for organizing the ECPA 2021. We hope that this special issue is useful to the reader looking for latest developments in the wide field of precision agriculture.

